# Development, Characterization, and In Vivo Evaluation of a Novel Aptamer (Anti-MUC1/Y) for Breast Cancer Therapy

**DOI:** 10.3390/pharmaceutics13081239

**Published:** 2021-08-11

**Authors:** Huma Khan, Vaidehi Makwana, Sofia Nascimento dos Santos, Carlos Eduardo Bonacossa de Almeida, Ralph Santos-Oliveira, Sotiris Missailidis

**Affiliations:** 1 Department of Life, Health and Chemical Sciences, Faculty of Science, The Open University, Walton Hall, Milton Keynes MK7 6AA, UK; huma.khan@bsigroup.com (H.K.); Vaidehi.makwana@syneoshealth.com (V.M.); 2 Radiopharmacy Department, Nuclear Energy Research Institute, São Paulo 05508-000, Brazil; snsantos@usp.br; 3 Division of Medical Physics, Radiation Protection and Dosimetry Institute, Brazilian Nuclear Energy Commission, Rio de Janeiro 22783-127, Brazil; cbonacos@ird.gov.br; 4 Nuclear Engineering Institute, Brazilian Nuclear Energy Commission, Rio de Janeiro 21941-972, Brazil; roliveira@ien.gov.br; 5 Laboratory of Radiopharmacy and Nanoradiopharmaceuticals, Zona Oeste State University, Rio de Janeiro 23070-200, Brazil; 6 Bio-Manguinhos Institute of Technology in Immunobiologics, Oswaldo Cruz Foundation, Rio de Janeiro 21040-900, Brazil

**Keywords:** cancer, aptamer, therapy, MUC1/Y, pharmacokinetics

## Abstract

MUC1, the transmembrane glycoprotein Mucin 1, is usually found to be overexpressed in a variety of epithelial cancers playing an important role in disease progression. MUC1 isoforms such as MUC1/Y, which lacks the entire variable number of tandem repeat region, are involved in oncogenic processes by enhancing tumour initiation. MUC1/Y is therefore considered a promising target for the identification and treatment of epithelial cancers; but so far, the precise role of MUC1/Y remains to be elucidated. In this work, we developed and identified a DNA aptamer that specifically recognizes the splice variant MUC1/Y for the first time. The DNA aptamer could bind to a wide variety of human cancer cells, and treatment of MUC1/Y positive cells resulted in reduced growth in vitro. Moreover, MUC1/Y aptamer inhibited the tumour growth of breast cancer cells in vivo. The present study highlights the importance of targeting MUC1/Y for cancer treatment and unravels the suitability of a DNA aptamer to act as a new therapeutic tool.

## 1. Introduction

The epithelial mucin 1 (MUC1) is a functional protein with important roles in the survival of cells. However, MUC1 is also distinctly associated with many malignancies, where several original characteristics of the protein are lost. As opposed to the typical expression, which is restricted to the apical surface of normal epithelial cells, MUC1 is found to be overexpressed in many adenocarcinomas, such as those of the breast, lung, ovary, pancreas, prostate, and numerous other epithelial organs. Other cancers also expressing MUC1 to a substantial degree include multiple myeloma, lymphoma and particular leukaemias [1]. Greater expression is due to the loss of polarisation. However, MUC1 displays many different features on tumour cells compared with the native protein, such as significantly reduced glycosylation due to the lessening number of tandem repeats present in the extracellular domain, combined with the extra addition of sialic acids, which also distort the cellular adhesion properties on malignant cells [2]. Interestingly, MUC1 can also operate as an adhesive molecule due to the attached carbohydrate structures, such as sialyl Lewis (a) and sialyl Lewis (x) in cancers of the pancreas and the colon [3]. 

Several MUC1 isoforms are generated through alternative splicing, and these are often found expressed within the same type of cells or tissues. The different alternative spliced variants vary in structure from the native MUC1 protein by lacking either the transmembrane domain, cytoplasmic domain, or the tandem repeat regions. Hence, each of the isoforms also differs in their functional properties, and most of the isoforms are overexpressed in cancer cells compared with cells obtained from benign tumours or normal epithelial cells [4]. 

The alternatively spliced variant MUC1/Y is identical to the MUC1 regarding the transmembrane domain and the cytoplasmic tail. However, this splice variant is lacking the tandem repeat region and the flanking region. This splice variant, like MUC1, possesses sequences that are also observed in cytokine receptors, suggesting that MUC1/Y can perform as a cytokine receptor and possibly be involved in similar ligand interactions [5]. Although MUC1/Y shares similarities with MUC1, the splice variant does not have a heterodimeric component, whereupon cleavage of the protein can occur [6]. Furthermore, this membrane-bound cell protein goes through transphosphorylation within the cell on the tyrosine and serine residues, which are part of the cytoplasmic domain; this is found to occur in breast malignancy upon binding to the MUC1/SEC protein [7,8]. The interaction between MUC1/Y and MUC1/SEC, which occurs at the extracellular domain, is not only thought to induce phosphorylation but is responsible for instigating many biological processes, such as changes to cell morphology and prompting of the signalling cascade. Triggers to initiate the signalling cascade are thought to occur via the interaction of the cytoplasmic phosphorylated residues to the GRB2 protein, a similar mechanism to that observed with MUC1. One of the many effects of the signal transduction produced by the MUC1/Y protein is the enhancement of tumour progression. Interestingly, in cancers of the breast, the expression of this splice variant is seemingly advantageous as MUC1/Y is apparently found to be present in malignant tissue yet undetectable in neighbouring normal tissue of the same organ, thus establishing MUC1/Y as a potential marker for identifying malignancy [6,9].

Obermair et al. [10] demonstrated the extensive expression of MUC1/Y after screening eight different cervical cancer cell lines, whereby seven out of eight were found to express this splice variant. Similarly, when investigating primary ovarian tumour samples, it was observed that MUC1/Y is more frequently expressed in malignant than in benign tumours. From the 55 ovarian cancer samples that were analysed, 54 of those samples expressed MUC1/Y, proposing that MUC1/Y is a strong candidate for indicating the presence of malignancy. Whereas the MUC1/Y splice variant is coexpressed with the splice variant MUC1/SEC in benign tumours, this is not the case in malignant tumours [4].

We previously selected aptamers against the tandem repeat peptide of the MUC1 glycoprotein [11] and the glycosylated form of MUC1 [12]. Additionally, we utilized such aptamers for diagnostic applications [12] and radiolabelled them for diagnostic and therapeutic applications [13,14,15]. Finally, we coupled these aptamers to nanoparticles to improve their biodistribution and tumour delivery properties [16,17,18]. In this work, we demonstrate, for the first time, the development of a DNA aptamer against a MUC1/Y alternative splice variant aiming to obtain novel biopharmaceutics with improved specificity for MUC1/Y positive cancers. We characterized these aptamers with a series of biochemical and biophysical techniques and evaluated them in preclinical models for their therapeutic potential.

## 2. Materials and Methods

### 2.1. Aptamer Targets—MUC1/Y Peptides: 10mer and 20mer

Since the MUC1/Y splice variant is a transmembrane protein, the full protein cannot be used in the selection procedure due to the complexities involved in its isolation and purification. Thus, two synthetic peptides were designed composed of amino acids flanking the splice site present in the MUC1/Y protein. The MUC1/Y peptides used as targets for selection consist of 10 and 20 amino acids, referred to as 10mer peptide and 20mer peptide, respectively. The 10mer peptide sequence (TEKNAFNSSL) was selected based on five amino acids before the splice site, and five amino acids after the splice site, distinguishing the MUC1/Y from the MUC1 extracellular domain protein sequence. The 20mer peptide sequence (SVPSSTEKNAFNSSLEDPST) is an extension to the 10mer peptide of five additional amino acids on either side; hence, 10 amino acids before and 10 amino acids after the splice site [19]. Both peptides were HPLC purified by the Oligonucleotide and Peptide synthesis unit of the Queen’s Medical Centre, University of Nottingham, UK. 

### 2.2. Single Stranded Aptamer Library and Primers

The SELEX single-stranded oligonucleotide library consists of a central random region of 25 nt flanked by primer hybridization sequences comprising 23 nt on one side and 24 nt on the opposite end. The library sequence used was 72 nt in length: 5′-GGGAGACAAGAATAAACGCTCAA (random 25 nt region) TTCGACAGGAGGCTCACAACAGGC–3′. The amplification primers are as follows: 5′-end forward primer GGGAGACAAGAATAAACGCTCAA & 3′-end reverse primer GCCTGTTGTGAGCCTCCTGTCGAA. The combinatorial oligonucleotide library and amplification primers were HPLC purified and obtained by the Oligonucleotide and Peptide synthesis unit of the Queen’s Medical Centre, University of Nottingham, UK.

### 2.3. Biotinylation of the MUC1/Y Peptides

The MUC1/Y peptides (10mer and 20mer) were labelled with biotin–xx using a FluoReporter^®^ biotin-XX Protein Labelling Kit (Invitrogen Molecular Probes, Paisley, UK). The biotinylation of the peptides was conducted according to the manufacturer´s protocol. The peptide and the biotin–xx SE (1:2 molar ratio, respectively) were incubated at room temperature for 2 h with gentle shaking. The labelled peptide reaction mixture was purified on a PD-10 (GE Healthcare, Chalfont St Giles, UK) desalting column using PBS (pH 7.2) to elute the fractions.

### 2.4. Selection of Aptamers

Aptamers were selected using a mixture of one-round selections on PCR tubes and a consecutive selection of 8 rounds using affinity chromatography, as follows:

#### 2.4.1. Single Round Selection on Streptavidin Coated Tubes

This single round selection protocol employs immobilized biotinylated peptides on streptavidin-coated PCR tubes. The streptavidin-coated PCR tubes have a binding capacity of 15 ng/tube for biotin. The immobilization of the peptides in each streptavidin coated PCR tube was conducted in a 1:2 ratio of the biotin binding capacity (15 ng) to the biotinylated peptides (30 ng) and incubated for 3 min at 37 °C. Upon completion of the incubation period, excess biotinylated peptides were removed. The aptamer library was incubated with the immobilized MUC1/Y 10mer and 20mer peptide at room temperature for an hour with gentle shaking. Upon completion of the incubation period, excess aptamer library was removed, followed by a single wash with a binding solution. Two variations were used in the elution protocol, based on increased salt concentration or increased temperature, to effect denaturation of the aptamer-peptide complex. *Salt Elution:* The elution of the aptamers was performed via washing each tube individually with increasing salt concentrations, using an initial wash with 0.2 M NaCl and further elution washes using 0.1 M increments of NaCl concentration to a maximum of 1.5 M NaCl. *Temperature Elution:* The aptamers were eluted by washing each tube individually with 100 µL sterile water at increasing temperatures following an initial wash with sterile water at 25 °C for 2 min. Subsequently, the incubated water was removed and transferred to a fresh tube. Further elution wash steps were conducted in steps from 25 °C to a maximum of 95 °C temperature, using 5 °C increments.

#### 2.4.2. Affinity Chromatography-Based Selection

The 20mer peptide was immobilised onto a Sepharose resin and packed into a 1 mL column. The amplified aptamer library in potassium phosphate buffer (KPB) was incubated with the peptide for 1 h at 37 °C (rotating continuously). The column was subsequently washed with KPB to eliminate unbound species, and bound aptamers were eluted in KPB with increasing salt concentrations from 1–1.5 M NaCl (0.1 M increments) and then a final wash with 3 M NaSCN. Following desalting, the fractions were amplified using unidirectional PCR and reloaded into the column in a series of 8 rounds of selection and amplification. At the last round, fractions were desalted, amplified, and visualised on a 2% agarose gel. Aptamers were observed up to the highest end of the gradient, the 3 M NaSCN fraction, and these were subsequently cloned and sequenced. Three aptamers resulted from this second selection step [11].

All selected aptamers were analysed for secondary structure, based on the mFold program, available online free-of-cost at the MFold Web Server of the University of Albany [20]. All the structures were predicted at 37 °C with 100 mM NaCl and 5 mM MgCl_2_.

### 2.5. Ru (II) Complex Displacement Assay

#### 2.5.1. Ru (II) Complex Binding to the Aptamer (in Binding Solution)

A stock solution of Ru (II) complex was titrated into 50 nM aptamer. Upon each aliquot, the emission spectrum (excitation wavelength: 434 nm) of the solution was recorded (until no spectral changes could be observed upon further additions). In order to quantify the binding, fluorescence changes at 610 nm were plotted as a function of Ru(phen)2.dppz.BF4 concentration. The data were best fit to a sigmoidal function from which a value for Kd was determined.

#### 2.5.2. Peptide (20mer) Binding to the Aptamer and Displacement of the Ru (II) Complex 

The individual aptamers (50 nM) were mixed with near saturating concentrations of the dye (determined from titration experiments: 900 nM for S11, 1.5 µM for S51 and 2 µM for S75) and incubated until no fluorescent changes could be observed (~30 min). Subsequently, a stock solution of the 20mer peptide was titrated into the aptamer-dye solution and the emission spectrum was recorded (until no further changes could be observed). The relative fluorescence decrease at 610 nm was plotted as a function of peptide concentration, and data could fit to a sigmoidal, hyperbolic or quadratic function equally well. The fluorescence of the 20mer was recorded to see if it emits at the same wavelength used for the assay (i.e., between 550–650 nm). The 20mer only slightly emits at 610 nm, which is insignificant compared with the fluorescence changes observed during the ruthenium assay titrations. The fluorescence of the peptide only becomes apparent when the aptamer becomes saturated with the peptide in excess, and all the dye is displaced. Although it is reported that the dye itself has no fluorescence when in a solution, we observed a small and relatively insignificant signal compared with that of the dye when it is intercalated into DNA; this was considered when assessing data from experiments following the titration of the dye into the aptamer, by subtracting the emission of the free dye from the intercalating fluorescence of the dye-aptamer complex.

### 2.6. Thermal Denaturation Assay

A mixture composed of the aptamer (0.180 mM) and either the 10mer or 20mer peptide (180 mM) in a 1:1000 ratio aptamer to the peptide in binding solution was incubated for 1 h at ambient temperature. Subsequently, the 10× SYBR green I nucleic acid gel stain (2.5 mL) was added to the reaction mixture and kept on ice (4 °C) for 30 min. The aptamer-peptide mixture was heated from 3 °C to 100 °C at a 1 °C/min rate (denaturation step), followed by a cooling down process from 100 °C to 3 °C at a 1 °C/min rate (renaturation step). The fluorescence emission was recorded every minute during the denaturation step; for every 10 °C, increments from 80 °C to 10 °C were also recorded and thereafter, at 5 °C and 3 °C. The denaturation-renaturation cycles were repeated in triplicates and ran on a DNA Engine Opticon 2 Real-Time PCR (MJ Research, Waltham, MA, USA.) at 520 nm using excitation at 497 nm. The final analysis of the data was conducted using the software Origin 6.0. (Origin Lab Corporation, Northampton, UK) The SYBR green I nucleic acid gel stain was purchased from Sigma–Aldrich (Dorset, UK).

### 2.7. Serum Stability Assay

Serum stability assays were performed for each aptamer in mouse and human serum, purchased from Sigma–Aldrich (Dorset, UK), using a DNase solution as the control. The assay consisted of incubating a 3 µM solution of each aptamer in serum (final concentration of 93%) or with 0.1 U/mL DNase at 37 °C. At designated time points, the reaction was quenched and frozen immediately in liquid nitrogen. PAGE analysis was used to visualise the degradation of the aptamers in time. The stability assays in human serum were initially performed over 4 h but were subsequently repeated over 24 h to acquire a complete picture of the aptamer stability.

### 2.8. Cell Culture

MCF-7 (ATCC: HTB-22, breast cancer cell line), DU145 (ATCC: HTB-81, prostate cancer cell line), Calu-6 (ATCC: HTB-56, lung cancer cell line), A498 (ATCC: HTB-44, kidney cancer cell line), SK-OV-3 (ATCC: HTB-77, ovarian cancer cell line), HT-29 (ATCC: HTB-38, colon cancer cell line), PC-3 (ATCC: CRL-1435), OVCAR-3 (ATCC: HTB-161, ovarian cancer cell line), A549 (ATCC: CCL-185, lung cancer cell line), and MDA-MB-231 (ATCC: HTB-26, breast cancer cell line) were grown at 37 °C and 5% CO_2_ in minimal essential medium Eagle (MEM) cell culture medium supplemented with 10% fetal bovine serum and, 1% L-glutamine. Trypsin-EDTA, minimal essential medium Eagle (MEM), L-Glutamine, fetal bovine serum and cell culture flasks (75 cm^2^, surface treated with filter cap) were purchased from PAA Laboratories (Somerset, UK). Hanks balanced salt solution was obtained from Sigma–Aldrich (Dorset, UK).

### 2.9. Fluorescence Activated Cell Sorting (FACs)

All cells used in the FACS analysis were washed with PBS. Fluorescently labelled (Cy3) aptamers in PBS were placed at 95 °C for 5 min and were subsequently cooled on the bench slowly. Cy3 fluorescently labelled aptamers (2 µM) were incubated with 500,000 cells in 200 µL of PBS at 4 °C for 1 h. After incubation, the cells were washed by centrifugation for 5 min at 1000 rpm with PBS (400 µL × 3) and resuspended in PBS (400 µL), aiming for a total cell count of either 5000 or 10,000 cells, for analysis on the flow cytometer. The analysis was performed on a FACS Calibur, Becton Dickinson flow cytometer (BD, San Jose, CA, USA) with a maximum sort rate of 10,000 total cells/second. Purchases of 5’ Cy3 labelled 25 oligonucleotides were from Integrated DNA Technologies (Budapest, Hungary). PBS tablets were purchased from Sigma–Aldrich (Dorset, UK).

### 2.10. Fluorescence Microscopy

MCF-7, DU145, CALU-6 and A498 cells (50,000 cells) were plated in duplicates on round glass coverslips, placed inside each well of a 24-well cell culture plate and grown overnight. The adhered cells were washed with PBS (100 µL), followed by incubation with aptamer (0.5 µM) in serum-free MEM (300 µL) per well for 3 h at 37 °C. Subsequently, the cells were washed with PBS (200 µL × 3), followed by the addition of 4% paraformaldehyde (PFA) (200 µL) and incubated for 15 min at room temperature. The cells were then washed with PBS (200 µL × 2), treated with 0.1% Triton (200 µL) for 10 min at room temperature, followed by a PBS wash (200 µL × 2). The cells were then incubated with Hoechst 33258 (75 µL of 1 mg/mL) for 15 min at room temperature, followed by a PBS wash (200 µL × 3). The glass inserts were mounted onto glass microscope slides using CFPVOH (polyvinyl alcohol) and AF100 anti-fadent (10:1 ratio). The slides were visualised under a BX6l motorized Olympus DP72 fluorescence microscope digital camera, using Cy3 and DAPI filter with 40× magnification. The images were analysed using the software ImageJ (U.S. National Institutes of Health, Bethesda, MD, USA).

### 2.11. Cell Viability Studies

SRB assays were performed using DU145, MCF-7 and A498 (negative control) tumour cell lines in 96-well cell culture plates. Cells were plated at 1000 cells/well for DU145 and MCF-7, and 2000 cells/well for the A498 cell line and were incubated at 37 °C overnight (the cell densities varied in the optimisation experiments, as stated in the results). Aptamers (in PBS) were at concentrations starting from 100 µM with subsequent two-fold dilutions across the plate (in media) and were incubated with the cells (in triplicate measurements) at 37 °C for 3 or 6 days (depending on the experiment). Prior to incubation, the aptamers were placed at 95 °C for 5 min and left to cool on the bench and filtered through a 0.2 µm filter unit. After the incubation period, the cells were fixed by removing the growth media and adding 10% TCA in fresh media, leaving the plates at 4 °C for 1 h. Plates were then washed with distilled water and left until completely dry. Subsequently, 0.4% SRB in 1% acetic acid (100 µL) was added to the wells and incubated for 30 min, followed by washing with distilled water, drying, and the addition of 100 µL of 20 mM Tris-HCl, pH 10.5. The absorbance at 564 nm for individual plates was immediately recorded.

### 2.12. In Vivo Studies

In order to evaluate the clinical application of the S11b DNA Aptamer against the MUC1/Y, preclinical studies in MDA-MB-231 tumour-bearing mice were conducted. NOD/scid mice were bred at the animal facility of IPEN (Brazil); all experiments complied with the relevant laws and were approved by the local animal ethics committee protocol approval 181/17 (date of approval 19 January 2017). Mice were housed in standard ventilated cages with controlled 12:12 h light/dark cycles, and provided unlimited access to water and standard rodent feed. All animal handling followed institutional guidelines. 

For tumour induction, 20 eight-week-old female NOD/scid mice were subcutaneously injected with 2 × 10^6^ MDA-MB-231 cells and growth was accompanied for 5 weeks. Mice were observed three times per week for evidence of distress, ascites, paralysis, or excessive weight loss. When the tumour reached approximately 350 mm^3^, mice were divided into 2 groups:

Group 1: A total of ten MDA-MB-231 tumour-bearing mice were treated intraperitoneally for 5 consecutive days with 100 mg/Kg of S11b DNA aptamer.

Group 2: Control group—A total of ten MDA-MB-231 tumour-bearing mice were treated intraperitoneally for 5 consecutive days with a saline solution (0.9% NaCl).

### 2.13. Pharmacokinetic of S11b DNA Aptamer against the MUC1/Y

The pharmacokinetics (PK) parameters: concentration at zero time, elimination constant (K), the volume of distribution, elimination of half-life and blood clearance were evaluated in healthy Wistar rats (n = 12). In order to perform the PK analysis, 200 µg of radiolabelled S11b DNA aptamer was injected retro-orbitally into healthy Wistar rats, and at times of 1, 2, 4, 8, and 24 h, 100 µL of blood was collected from the tail vein. The conversion of radioactive activitye to mass of S11b DNA aptamer against the MUC1/Y was calculated considering the initial mass (200 µg) administered and the labelling quality control data using the radioactive decay formula: $N=N_{o}e^{-ƛt}$.

### 2.14. Radiolabelling of S11b DNA Aptamer against the MUC1/Y with ^99m^Tc

The radiolabelling process was done using 150 µg of S11b DNA aptamer against the MUC1/Y incubated in stannous chloride (SnCl_2_) solutions (80 µL/mL) (Sigma–Aldrich, St Louis, MO, USA) for 20 min at room temperature. Then, this solution was incubated with 100 µCi (approximately 300 µL) of technetium-99m (^99m^Tc) for another 10 min. In order to establish the quality control of the labelling process, radio thin layer chromatography (RTLC) was performed using Whatman paper no.1 as the stationary phase, 200 µL of 99mTc- S11b DNA aptamer against the MUC1/Y as spot point and acetone (Sigma–Aldrich, St Louis, MO, USA) as the mobile phase. The radioactivity of the strips was verified in a γ-counter (Perkin Elmer Wizard^®^ 2470, Shelton, CT, USA). The RTLC was performed in triplicate for each time point (0, 2, 4, and 6 h).

## 3. Results

### 3.1. Selection of Aptamers

For the selection of aptamers, the peptides 10mer and 20mer were initially immobilised on functionalised PCR tubes for a single selection cycle to eliminate lower binding or non-specifically bound aptamers, eluting with a salt gradient or temperature alterations. This round resulted in 40 aptamer sequences (data not shown), and the pool was used for subsequent rounds of selection on an affinity chromatography protocol against 20mer peptide. Elution was achieved with a NaCl gradient up to 1.5 M, with a final elution of 3 M NaSCN. Sequencing the aptamers from the 3 M NaSCN elution, we observed sequence consensus with the identification of three potentially high binding aptamers (S11, S51, and S75, Table 1), which were also present in the 40 sequences obtained from the initial single-round selection protocols, both against the 10mer and 20mer peptides; this indicated that these three aptamers bind to both the 10mer and 20mer peptides of MUC1/Y.

### 3.2. Predicted Structures of the Aptamers and Their Truncated Forms

**Figure 1:**
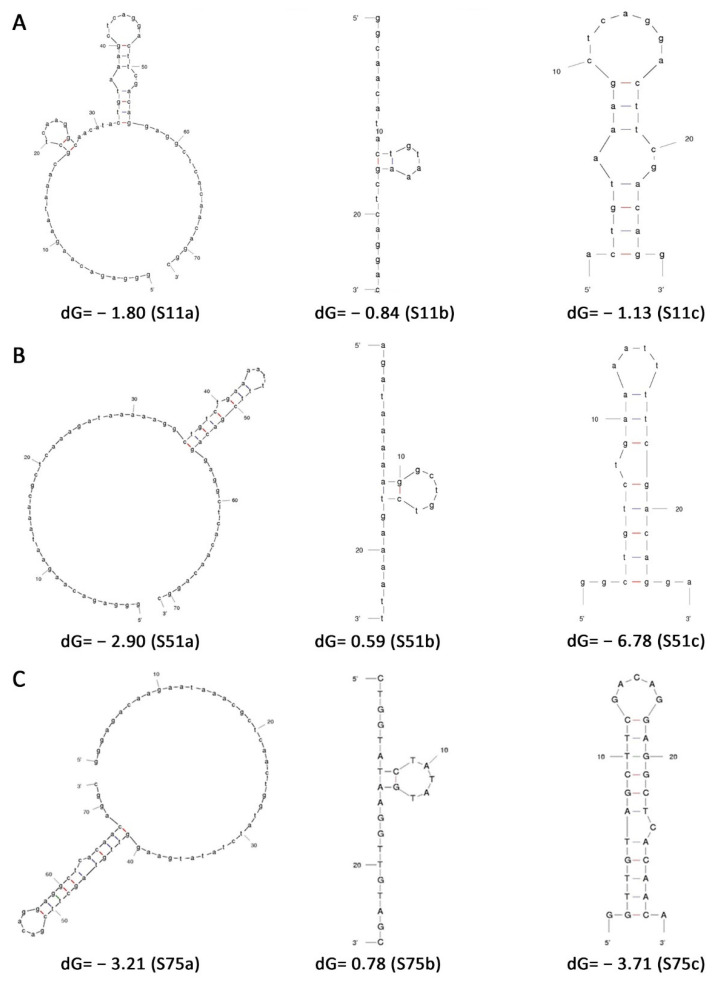
The three selected aptamers, (**A**) S11, (**B**) S51, and (**C**) S75 in three presentations: entire aptamer (**left**), variable region (**middle**), and structured region (**right**). All dG values are expressed in Kcal/mol.

For potential commercial use and cost reduction in synthesis, aptamers are often truncated to the sequence that dictates binding. In order to reduce the length of our aptamers from the full-length construct of 75 bases, we had the option to either truncate them by removing the two known primer sequences from either end and leaving only the selected 25 base sequences or to truncate the aptamer based on its predicted structural features. As we have no experimental information on the structure of the aptamer (X-ray or NMR) to determine which region dictates binding to the peptide, we used an oligonucleotide secondary structure prediction program (m-fold) to identify any potential structures that may form the aptamer binding domain. The predicted structures for each aptamer showed a common hairpin motif, implying that this may form the binding site of each aptamer. The structural results from the m-fold prediction program are shown in Figure 1.

We generated three constructs for each of the three lead aptamers, the full-length aptamers, the aptamers consisting of the variable region determined after the selection, and the region identified by m-fold to contain the hairpin motif. The different constructs used for further analysis were as follows: (i) full-length 75 bp aptamers (S11a, S51a, S75a); (ii) aptamers only consisting of the central variable sequence (S11b, S51b, S75b); (iii) part of the aptamer that forms the hairpin loop (S11c, S51c, S75c) (Figure 1).

### 3.3. Ru (phen)_2._ dppz.BF_4_ Displacement Assay

An alternative to measuring the binding interaction between aptamers and peptides, without directly labelling the aptamer, was to exploit the use of a DNA intercalator, Ru(phen)2.dppz.BF_4_. The unique advantage of using this dye compared to other DNA intercalators is the fact that when the dye is free in a solution, it has little or virtually no fluorescence, but when intercalated with dsDNA, it emits an intense fluorescence. By exploiting this property, we measured the interaction between the aptamer and peptide whereby the aptamer and dye would be mixed, and the fluorescence of the complex measured, yielding a strong signal. Therefore, it was expected that upon adding the peptide to the aptamer-dye complex, the peptide would bind to the aptamer and lead to the concomitant displacement of the dye from the aptamer, thus resulting in a decrease in fluorescence. These changes in fluorescence could then be used to measure and quantify the interaction between the peptide and aptamers by using a titration displacement assay. Although our aptamers are single-stranded, they are most likely to adopt secondary structures (as inferred from folding predictions established by *m*-fold (Figure 1), which would permit intercalation of the dye. Since the dye is commercially unavailable, it was necessary to synthesise it in house. Details of the synthesis have been previously reported [21]. Product identification, Ru(phen)2.dppz.BF4, was verified using NMR and mass spectrometry analysis (data not shown). 

Fluorescence measurements were performed by titrating the dye to an aptamer solution (50 nM) to estimate how much of the dye was needed to saturate the aptamer. Titration of the dye in the aptamer resulted in a significant increase in fluorescence compared with the free dye or aptamer alone; this indicates that the aptamers do adopt some degree of secondary conformation for the dye to intercalate. Fluorescence changes were plotted as a function of dye concentration, and fitting of the data allowed calculation of the *K*d for the Ruthenium-aptamer interaction of 0.35 µM for S11, 0.44 µM for S51, and 0.24 µM for S75 (Supplemental Figure S1).

**Figure 2:**
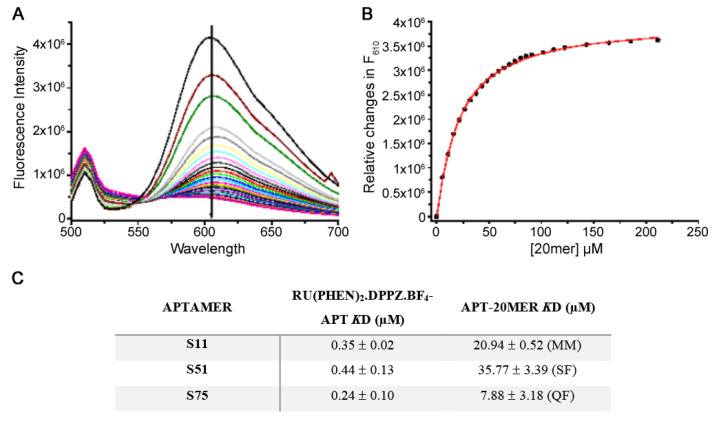
Fluorescence changes observed upon titration of the 20mer into the S11 aptamer-dye complex. (**A**) Emission spectra; (**B**) Fluorescence changes plot as a function of 20mer concentration. All experiments were performed in duplicate and the results presented are the average of these measurements; (**C**) Dissociation constants obtained from the Ru (II) complex displacement assay. Data for the aptamer-dye complex were all fit to a sigmoidal function while data for the aptamer-peptide complex best fit to differing functions: MM = standard hyperbolic function described by the Michaelis–Menten equation; SF = sigmoidal fit; and QD = quadratic function for binding.

To assess the binding of the 20mer peptide to the aptamers, the 20mer peptide was titrated into a solution of each of the aptamers (50 nM) and saturating concentrations of the dye. Titration of the peptide to the aptamer-dye complex produced a decrease in fluorescence, indicating that the dye becomes displaced from the aptamer and inferring that the peptide binds to the aptamer, causing this displacement. Fluorescence changes were plotted as a function of peptide concentration, and fitting of the data provided a displacement *K*d for the aptamer-peptide complex (Figure 2A–C). All three aptamers (S11a, S51a and S75a) were analysed by the ruthenium displacement assay in their full version. However, with smaller versions (25b) of each aptamer, due to small size and reduced base-pairing efficiency, intercalation resulted in a small fluorescence signal that could not be used for affinity calculations. The aptamer S11 with a Kd of 20.94 µM for the 20-mer peptide, which was also the only aptamer identified in all the selection procedures tested and demonstrated a robustness of binding to the peptide independent of the selection conditions, was chosen for all subsequent experiments. The value of the Kd calculated is indirect, and the high value may be due to the positioning of the peptide in respect to the aptamer and not a direct measurement of affinity, as this is not a constant of dissociation but of displacement.

However, we used the same experiment to demonstrate the aptamer specificity, testing our aptamers against the Variable Tandem Repeat (VTR) sequence of the MUC1 and a non-related protein (lipase). We showed that at least 100-fold more MUC1 peptide was necessary to displace the Ru (II) complex, whereas lipase was not capable of displacing the Ru (II) complex up to the concentration tested (250 µM) (Supplemental Figure S2).

### 3.4. Thermal Denaturation Assay

Determination of the melting temperature can be employed to assess the effects of any modifications made to a nucleic acid or an interaction with a ligand in terms of stabilization or destabilization of the secondary structure of this nucleic acid.

**Figure 3:**
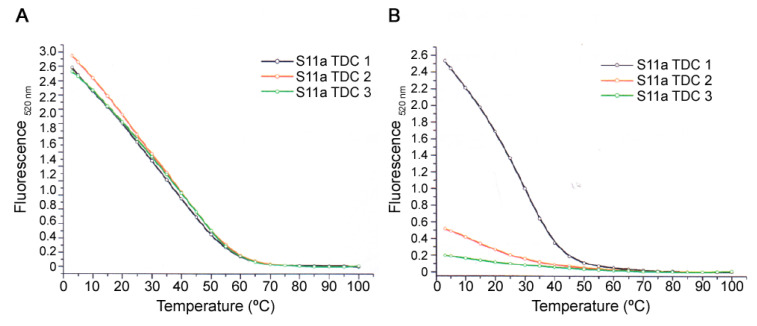
Thermal denaturation of S11a. (**A**) Fluorescence melting profiles of S11a alone at 3 TDC; (**B**) Fluorescence melting profiles of S11a with the 20mer peptide at 3 TDC.

In order to determine the thermal denaturation of the MUC1/Y aptamers, the fluorescence analysis approach was employed using the HRM SYBR green dye, as this technique is considerably more sensitive than the UV absorbance method. Each aptamer of the S11 series (S11a, S11b and S11c) was incubated with the peptides (10mer and 20mer), short peptides without any secondary or tertiary structure, in a 1:1000 molar ratio, in a binding solution for 1 h at ambient temperature. Upon incubation, the SYBR green dye was added to the aptamer solution and incubated for 30 min on ice. Thermal denaturation cycles (TDCs) were performed three times from 3 °C to 100 °C at a rate of 1 °C/min with a gradual renaturation process between each cycle. The emission was detected at 520 nm when excited at 497 nm. Figure 3 shows plots for S11a (A) and S11a with the 20mer peptide (B) at each TDC. In the case of S11a (Figure 3A), minimal variation in fluorescence was observed between the three TDC. When S11a was incubated with the 20mer peptide (Figure 3B), on the other hand, the fluorescence value at the beginning of the analysis was slightly lower compared with the free aptamer (Figure 3A). However, dramatic changes in fluorescence were observed during the second and third TDCs, where the fluorescence values at the beginning of the cycle were 0.5 and 0.2, respectively. This effect suggests that the peptide inhibits the renaturation of S11a, resulting in the aptamer remaining mainly in an open conformation. A similar trend of a gradual decrease in fluorescence through each cycle was also observed when the 10mer peptide was used (data not shown).

**Figure 4:**
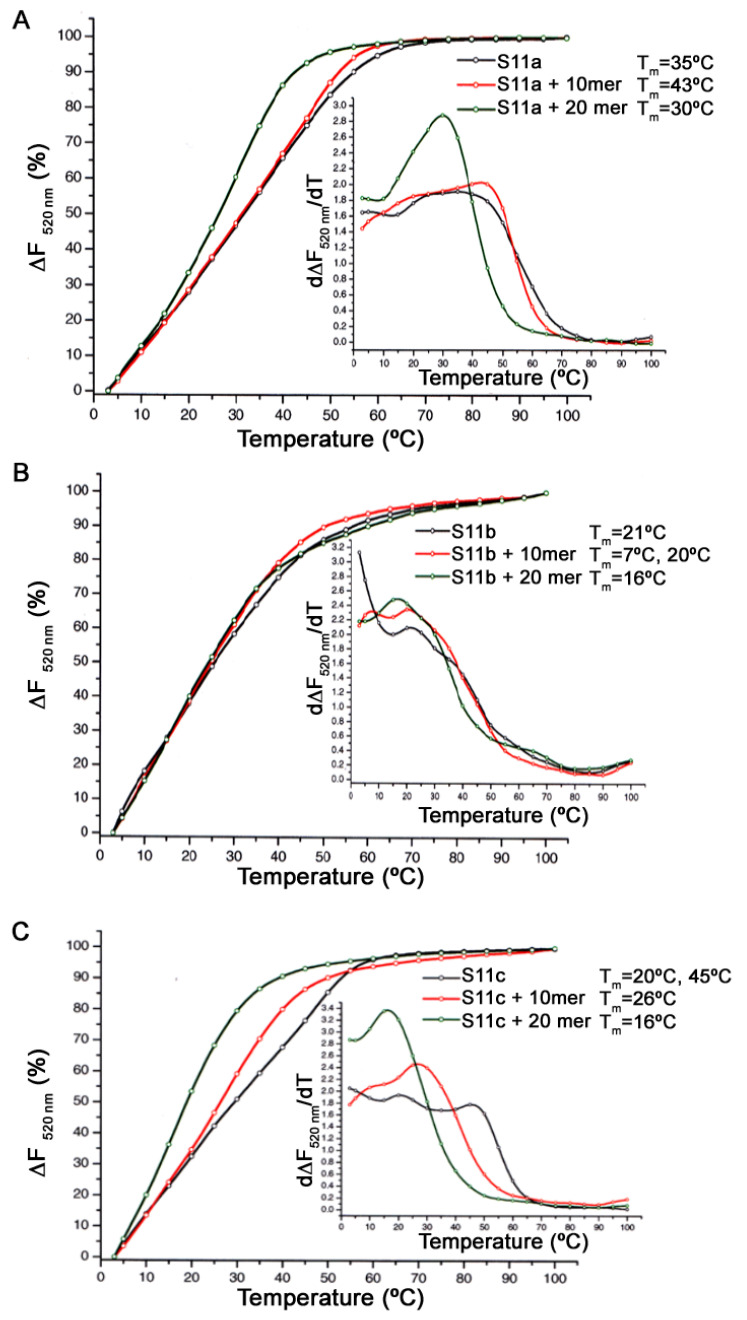
Thermal denaturation of (**A**) S11a, (**B**) S11b, and (**C**) S11c. Comparison of the fluorescence melting profiles of S11a, S11b and S11c with the 10mer and 20mer peptides.

In order to determine the effect that 10mer and 20mer peptides have on the conformation of the S11a, S11b and S11c aptamers, and thus its significance upon binding, the fluorescence melting profiles were evaluated and presented as the percentage change in fluorescence (∆F_520nm_)(%) vs. temperature. The melting temperatures (T_m_) was determined as the first derivative. The addition of the 10mer peptide increased the Tm of the S11a aptamer from 35 °C to 43 °C, whilst the addition of the 20mer peptide reduced the T_m_ by 5 °C down to 30 °C (Figure 4A). Figure 4B,C show the fluorescence melting profile of the truncated aptamers (S11b and S11c) with the MUC1/Y peptides. The addition of the 10mer peptide to S11b causes a change in the melting profile as two T_m_ are observed (7 °C and 20 °C). Although there is no significant difference between the later T_m_ and the free S11b (21 °C), the 20mer peptide reduces the T_m_ to 16 °C (Figure 4B). The addition of the 10mer and 20mer peptides to S11c causes the melting profile to change, providing a single Tm of 26 °C and 16 °C, respectively, from S11c (20 °C and 45 °C). If the later T_m_ of S11c (45 °C) is considered, then the addition of the 10mer peptide decreases the thermostability of the aptamer by ca. 20 °C, whilst the 20mer causes a 30 °C decrease (Figure 4C). Thus, the MUC1/Y peptides have the greatest effect on the melting temperature of the S11c aptamer compared with the S11a and S11b.

### 3.5. Serum Stability Assay

The stability of the aptamers was investigated in mouse and human serum. Each of the aptamers was incubated in the serum and a sample was removed at designated time points from the incubated solution; the reaction was then quenched and frozen. Analysis of aptamer degradation was conducted using PAGE analysis. Stability assays were performed with all three aptamers in their complete sequence. The results of the assay demonstrated that all the aptamers were more stable in human serum compared with mouse serum (Supplemental Figure S3—data for S11 are shown as a representation). Although aptamers do not need any base modification for clinical studies or actual treatments in humans, they would present reduced stability in mice. To address this, we also used an added inverse thymine base at the end of the aptamers, which extended the lifetime of the aptamers in mice serum, with no digestion observed in the first 24 h.

#### 3.5.1. MUC1/Y Aptamers S11b and S11c Bind to Cancer Cells but Only S11b Is Cytotoxic In Vitro

**Figure 5:**
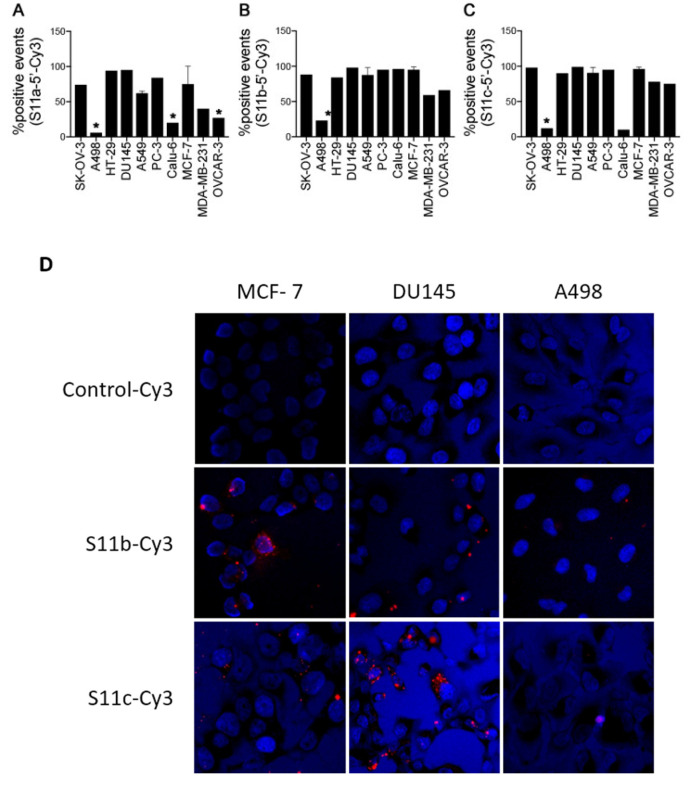
(**A**–**C**) Percentage of positive events of MUC1/Y in SK-OV-3, A498, HT-29, DU145, A549, PC-3, Calu-6, MCF-7, MDA-MB-231 and OVCAR-3 using the aptamers (**A**) S11a, (**B**) S11b, and (**C**) S11c. (**D**) Representative immunofluorescence of MCF-7, DU145, Calu-6, and A498 cells stained with control-Cy3 (aptamer SP68; selected against a different/non-MUC1 tumour marker peptide), S11a-Cy3 or S11b-Cy3, * *p* < 0.05.

In order to assess the binding of the S11 aptamers to the full-length biomarker and investigate which cancer cell lines express MUC1/Y, flow cytometry was performed. Each of the full length and shorter aptamers was labelled with 5′Cy3 and incubated with a panel of cancer cells in PBS for 1h on ice. Following incubation, the cells were washed and then analysed for binding events, compared to a control consisting of cells only. The percentage of positive binding events determined for each cell line is summarised in Figure 5A–C. Aptamers demonstrated binding to cell lines expected to express MUC1/Y (such as MCF-7, PC-3, and DU145) [22,23] and provide new information about cell lines with unknown expression patterns of MUC1/Y. Shorter aptamers (S11b and S11c) demonstrated higher cell binding than their full-length counterparts, possibly due to the steric hindrance of primers that do not actively participate in the binding event.

We then evaluated the aptamer interaction with MUC1/Y by fluorescence microscopy on two cell lines where the aptamers showed the best binding, namely DU145 and MCF7. As a control, we used A498 cells, which were shown to be negative for MUC1/Y. The assays were only conducted with the shorter versions of aptamer S11 (S11b and S11c versions), as these were deemed clinically relevant. As shown in Figure 5D, Cy-3-labeled S11b and S11c aptamers are bound to DU145 and MCF7, whereas no binding was observed in A498 cells.

We further evaluated the survival of tumour cell lines treated with our selected MUC1Y aptamers. In the initial evaluation, DU145 and A498 cell lines were treated for 6 days with the S11b and S11c aptamers. The S11c aptamer did not show any cell toxicity in either of the cell lines tested. However, significant cell toxicity was observed in the DU145 cells using the S11b aptamer (IC50 of 8.7 ± 0.28 µM) compared with the control (MUC1 aptamer) treated cells where no toxicity was found. As expected, A498 showed no evidence of cell kill occurring, which agrees with the flow cytometry data since this cell line does not express MUC1Y and is used as a negative control. Furthermore, the S11b aptamer was used as a monomer and dimer for treating MCF7 breast cancer cell lines to verify the potential improvement in the time or IC50 values. Both the monomer and dimer S11b aptamer showed cytotoxicity on MCF7 tumour cell lines. However, whilst the dimer demonstrated activity at both 3 and 6 days, the values were an order of magnitude higher (IC50 MCF7 3 days of 61.4µM and IC50 MCF7 6 days of 33.4 µM) than those obtained by the monomer in the 6-day study (IC50 of 4.81 ± 1.04 µM). Thus, the S11b monomeric aptamer was chosen for subsequent in vivo experiments.

#### 3.5.2. MUC1/Y Aptamer Inhibits Tumour Growth in a Mouse Model of Breast Cancer

**Figure 6:**
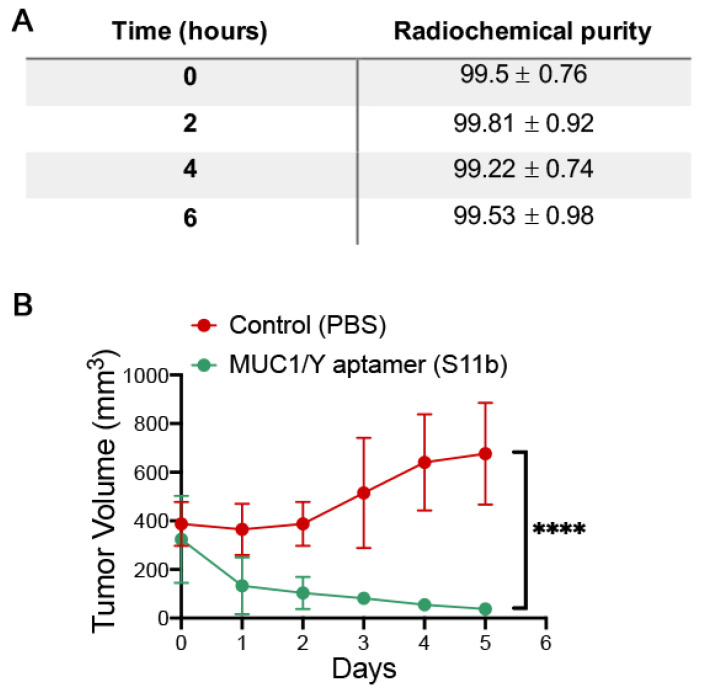
(**A**) Radiochemical purity of S11b-^99m^Tc aptamer at 0, 2, 4 and 6 h post-labelling in saline. (**B**) Tumour growth curve of MDA-MB-231 tumour-bearing NOD-scid mice control (red) and treated with 100 mg/Kg of S11b aptamer (green). Data are the mean (S.D.), *n* = 5. Data were analysed by a non-linear regression model. **** *p* < 0.05.

To evaluate the effects of S11b aptamer in animals bearing MDA-MB-231 tumours, we initially determined its pharmacokinetic properties using noncompartmental analysis. S11b was initially radiolabelled with ^99m^Tc, yielding a radiochemical purity >99% and stable for at least 6 h in saline (Figure 6A). S11b was also found to be stable in mouse and human serum for at least 4 h (Supplemental Figure S3).

For the pharmacokinetics studies, 200 µg of S11b-^99m^Tc aptamer were injected retro-orbitally into healthy rats and blood samples were obtained after 1-, 3- and 6-h post-injection and the radioactivity was measured. It was found that the volume of distribution (Vd), which estimates the distribution of S11b-^99m^Tc aptamer into extravascular tissues (tissue deposition) [24], was 0.00324 mg/L, suggesting that a high amount of S11b-^99m^Tc aptamer still remained in the plasma and was poorly distributed into the body during the period of time evaluated. The blood clearance (CL) of S11b-^99m^Tc aptamer (an essential value to evaluate the elimination rate) [25,26] was found to be 0.00025 mg/L/hour, suggesting that S11b-^99m^Tc aptamer is slowly eliminated from the blood circulation. The half-life (t 12) of S11b-^99m^Tc aptamer, which represents the time to purge 50% of the S11b-^99m^Tc aptamer from plasma, was 0.402 h (~24 min), which allows the aptamer to reach the tumour.

We finally assessed whether S11b aptamer could have therapeutic potential in vivo. Therefore MDA-MB-231 cells were subcutaneously inoculated in NOD-scid mice. When tumours reached approximately 350 mm^3^, 100 mg/Kg of S11b DNA aptamer or saline (control group) was intraperitoneally administered once a day for a total period of five days. Surprisingly, observed a statistically significant reduction of approximately 88% in the tumour volume of S11b-treated mice compared with the control group (Figure 6B).

## 4. Discussion

The MUC1 gene encodes for a transmembrane glycoprotein overexpressed and aberrantly glycosylated in several types of epithelial cancers, which plays an important role in the progression of the disease [27]. Tumour-associated MUC1 differs from that expressed in healthy cells by presenting increased sialylation and loss of core 1 O-glycans and abnormal cellular distribution [28,29]. These features are responsible for tumour cell loss of polarity, redistribution of cell surface growth receptors such as EGFR and hyperactivation of a critical signalling pathway that drives tumour progression [30].

Several isoforms of MUC1, such as MUC1/A, MUC1/B, MUC1/C, MUC1/D, MUC1/X (or MUC1/Z), MUC1/Y, and MUC1/ZD, are discovered in humans as a result of alternative splicing, exon skipping, and intron retention. MUC1/Y, in particular, was found to be highly expressed in breast, ovarian, and prostate cancer cells [9,23]. MUC1/Y has been implicated in several cellular functions, such as the overexpression of cytokines and the modulation of immune responses, through its binding with MUC1/SEC. Furthermore, MUC1/Y has been shown to induce the transcription of proinflammatory cytokines via NF-kB [8,9,31].

In this work, we selected aptamers against the MUC1/Y alternative splice variant and characterized them for their stability, binding affinity, capacity to bind to cell lines expressing MUC1/Y, and as tools to determine the presence of MUC1/Y in cell lines with unknown expression patterns. Furthermore, with the intention of developing such aptamers as potential anticancer agents, we tested their capacity to inhibit tumour cell growth in vitro and in vivo. As a result, we demonstrated for the first time a potential role of MUC1/Y in cancer growth and proliferation in a mouse model of breast cancer and its potential as a target for anticancer therapeutics. Even though we used only the exposed peptide at the splice region of the MUC1/Y as the target and selected aptamers showed a relatively low affinity in terms of their Ru (II) complex displacement constant towards the peptide, they were capable of binding to cancer cell lines in flow cytometry and fluorescent microscopy experiments. Furthermore, these aptamers could reduce tumour cell growth both in vitro and in vivo in a MDA-MB-231 tumour-bearing mice model.

A previous demonstration showed that the interaction of MUC1/Y with MUC1/SEC could enhance second messenger proteins capable of eliciting pro-tumoural cellular responses [9]; this may indicate that blocking MUC1/Y inhibits its interaction with MUC1/SEC and its binding to tumour cells, inhibiting their proliferation. Additionally, blocking the interaction of MUC1/Y with MUC1/SEC could have an anti-inflammatory effect, reducing tumour-associated inflammation through the blocking of proinflammatory cytokine expression.

Among all therapeutic molecules, DNA aptamers have increasingly drawn the attention of researchers due to their ability to stably bind a wide variety of molecules. DNA aptamers can be more specific than small molecules and peptides, but they are smaller and more flexible than antibodies or antibody fragments. Aptamers have a higher tissue penetration capacity than antibodies because of their smaller size; they are more effectively eliminated from the bloodstream and promote little or no immunogenicity [32,33]. MacugenTM remains the only aptamer approved by FDA (2004) to date with a therapeutic function, and its use is limited because of its choice of target, compared with Avastin. However, aptamers remain molecules of high therapeutic potential, with the nature of a biologic but production of a synthetic molecule. Our present study highlights their potential in the treatment of MUC1/Y-expressing tumours, as well as unveils the role of MUC1/Y in cancer growth and its potential as a target for cancer therapy.

## Figures and Tables

**Table 1:** Sequences of the aptamers eluted under stringiest conditions in all selection conditions against both targets.

Name of Aptamer	Length of Aptamer	Sequence of Aptamer
S11	72 nt	gggagacaagaataaacgctcaaggcaacatactgtaaagctcaggacttcgacaggaggctcacaacaggc
S51	71 nt	gggagacaagaataaacgctcaaagataaaaaggctgtctgaaaattttcgacaggaggctcacaacaggc
S75	72 nt	gggagacaagaataaacgctcaactggtatctatatgaaggttgtagcttcgacaggaggctcacaacaggc

## Data Availability

Data is contained within the article or supplementary materials.

## Supplementary Materials

The following are available online at https://www.mdpi.com/article/10.3390/pharmaceutics13081239/s1, Figure S1: Ruthenium dye titrated into stock solution of aptamer. (A) Emission spectra of the titration (B) Fluorescence changes plot as function of the dye concentration. Figure S2: (A) The titration of Aptamer S11b with the VTR MUC1 sequence, where it takes a 100-fold more peptide to displace the ruthenium from this aptamer sequence, compared to the one required from the target MUC/Y peptide.(B) The effect of increasing concentrations of lipase on the fluorescence of free ruthenium is shown. Lipase was not shown to have an effect on the fluorescence of free ruthenium. (C) The effect of increasing concentrations of lipase on the fluorescence of ruthenium bound to an aptamer against MUC1/Y was investigated. Lipase showed no significant effect on the florescence of the aptamer-bound ruthenium, indicating no significant binding between the aptamer (variable region of Aptamer) and the protein, Figure S3: PAGE analysis of S11b degradation in mouse (A) and human serum (B).
