# Supplementary material for: Development, Characterization, and In Vivo Evaluation of a Novel Aptamer (Anti-MUC1/Y) for Breast Cancer Therapy
Source: Pharmaceutics. 2021 Aug 11;13(8):1239. doi: 10.3390/pharmaceutics13081239 (PMC8400696; doi:10.3390/pharmaceutics13081239)
Supplement: Supplementary file 1 [file pharmaceutics-13-01239-s001.zip › pharmaceutics-1317555-supplementary.pdf]

pharmaceutics
MDPI

Supplementary Materials: Development, Characterization, and In Vivo Evaluation of a Novel Aptamer (Anti-MUC1/Y) for Breast Cancer Therapy

Huma Khan, Vaidehi Makwana, Sofia Nascimento dos Santos, Carlos Eduardo Bonacossa de Almeida, Ralph Santos-Oliveira and Sotiris Missailidis

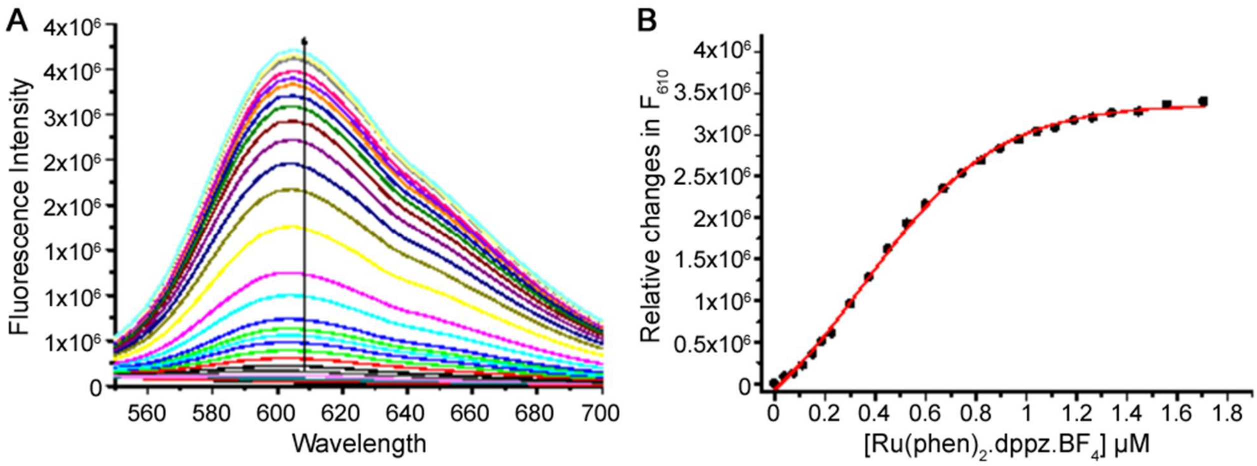

Figure S1. Ruthenium dye titrated into stock solution of aptamer. (**A**) Emission spectra of the titration (**B**) Fluorescence changes plot as function of the dye concentration.

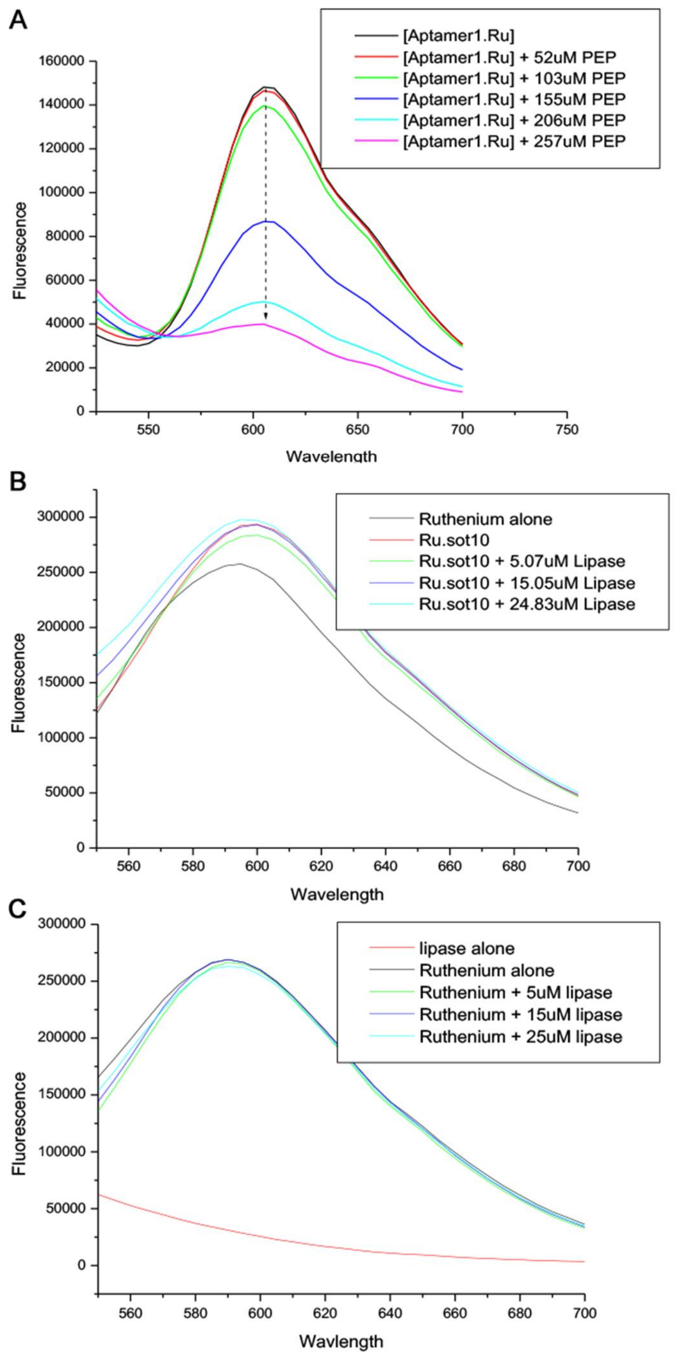

Figure S2. (**A**) The titration of Aptamer S11b with the VTR MUC1 sequence, where it takes a 100-fold more peptide to displace the ruthenium from this aptamer sequence, compared to the one required from the target MUC/Y peptide.(**B**) The effect of increasing concentrations of lipase on the fluorescence of free ruthenium is shown. Lipase was not shown to have an effect on the fluorescence of free ruthenium. (**C**) The effect of increasing concentrations of lipase on the fluorescence of ruthenium bound to an aptamer against MUC1/Y was investigated. Lipase showed no significant effect on the florescence of the aptamer-bound ruthenium, indicating no significant binding between the aptamer (variable region of Aptamer) and the protein.

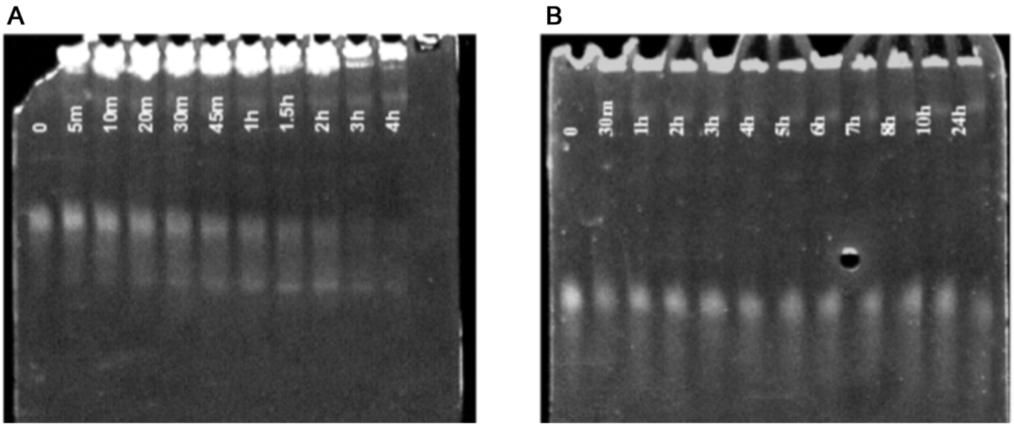

Figure S3. PAGE analysis of S11b degradation in mouse (**A**) and human serum (**B**).
